# SVF-GEL Cryopreserved for Different Times Exhibits Varied Preservation and Regeneration Potential After Transplantation in a Mouse Model

**DOI:** 10.1007/s00266-022-03065-5

**Published:** 2022-09-08

**Authors:** Yue Tao, Zheng-Nan Zhao, Xin-Jian Xiang, Ze-Xu Liang, Yu Zhao

**Affiliations:** Department of Plastic Surgery, First Affiliated Hospital of Anhui Medical University, 218 Jixi Road, Hefei, 230022 Anhui People’s Republic of China

**Keywords:** ASCs, Cryopreservation, Fat grafting, Stromal vascular fraction gel

## Abstract

**Background:**

Matrix vascular component (SVF) gels derived from fat preserve tissue integrity and cell viability under cryopreserved conditions, making them easy to inject again for later use. Here, we compared the preservation power and regeneration potential of SVF-gel under different cryopreservation times.

**Methods:**

The SVF-gel stored under − 20 °C, without cryoprotectant cryopreservation for 5, 15, and 45 days, with fresh SVF-gel as control. We evaluated the rate of volume retention after thawing the SVF-gel and the apoptosis rate of adipose-derived stem cells. Next, we analyzed retention rated, adipogenesis, angiogenesis, and connective tissue hyperplasia of the grafts, one month after subcutaneously transplanting the specimen into immunodeficient mice.

**Results:**

SVF-gel cryopreserved for 5 and 15 days exhibited no significant different in apoptosis rates relative to the control group. Extending the cryopreservation time to 45 days resulted in significantly increased and decreased apoptosis and volume retention rates of SVF-gel, respectively. SVF-gel grafts cryopreserved for 5 and 15 days exhibited no significant differences from those in the control group, although their weights and volumes still fluctuated. Extending the cryopreservation time to 45 days resulted in significantly decreased retention rates of the grafts. Histologically, extending freezing time resulted in a gradual decline in the graft’s health adipose tissue, as well as decreased angiogenesis, and connective tissue hyperplasia.

**Conclusion:**

Simple freezing of SVF-gel at − 20 °C conferred them with sufficient cell viability. Notably, short-term cryopreservation did not significantly increase the apoptosis rate, and it still had a certain regeneration after transplantation. However, prolonging freezing time to 45 days resulted in increased apoptosis rate and worsened transplantation effect.

**No Level Assigned:**

This journal requires that authors assign a level of evidence to each submission to which Evidence-Based Medicine rankings are applicable. This excludes Review Articles, Book Reviews, and manuscripts that concern Basic Science, Animal Studies, Cadaver Studies, and Experimental Studies. For a full description of these Evidence-Based Medicine ratings, please refer to the Table of Contents or the online Instructions to Authors www.springer.com/00266.

## Background

Autologous fat is considered an ideal soft tissue filling material in the field of repair and reconstruction, as well as cosmetic surgery, due to its rich content in the human body, coupled by several advantages such as conveniency, cost effectiveness and high biocompatibility. Since Coleman reported that his fat transplantation technology showed excellent transplantation results in 1995 [1], numerous studies have been conducted on fat transplantation [2]. Numerous studies have demonstrated that adipose-derived stem/progenitor cells (ASCs) attached to the extracellular matrix (ECM) contribute to the regeneration of newly formed adipose tissue [3, 4]. In 2017, Lu [5] first invented and reported a simple mechanical method that could not only remove selective removal of most mature cells from the adipose tissue but also enrich ASCs and natural extracellular matrix (ECM). They called the product of this process SVF-gel (stromal vascular fraction gel). Notably, SVF-gel has better retention rate and greater tissue integrity after transplantation and has shown superior efficacy to traditional fat transplantation during treatment of facial rejuvenation as well as volume expansion [6, 7].

However, in clinical practice, fat transplantation still needs multiple injections to achieve the desired effect. This phenomenon has inspired many researchers to study cryopreservation. Masanori Ohashi studied application of frozen fat for treatment of facial rejuvenation, increased breast volume and scar hyperplasia in 219 patients, and found that it was efficacious during continuous short-term follow-up. To date, however, no study has reported the effect of frozen fat grafters on the rate of volume retention during longer follow-up periods [8, 9]. Moreover, frozen fat face injection has been found to have a similar clinical effect to fresh facial fat injection [10]. Cryopreservation of adipose tissue is accompanied by various problems, key among them cooling and thawing temperatures, as well as the use of cryopreservation agents [11]. In fact, numerous controversies still exist due to the consideration of activity and safety [12–14]. In addition, studies have shown that most fat cells are killed during freezing and thawing, and the graft remains dead tissue, including oil sacs and fibrous tissue, phenomena that pause a risk of serious complications [15].

Previous studies have shown that adipose-derived stem/progenitor cells (ASCs) can not only survive cryopreservation [16] but also retain the ability to proliferate and differentiate into adipocytes, osteoblasts and chondrocytes [17, 18]. However, ASCs can only be obtained after digestion of adipose tissue with collagenase for 30–60 min, which increases the risk of contamination by unknown foreign substances and unwanted biomaterials [19]. In addition, adherent culture and purification of ASCs not only require special experimental equipment, but also takes a long time. Collectively, these factors limit the application of ASCs. Studies have demonstrated that Ascs-enriched SVF-gel can maintain tissue integrity and cell viability under cryopreservation. Moreover, they provide a longer retention rate than cryopreservation fat [20], hence can be applied in the cryopreservation of adipose tissue. Further explorations are needed to unravel more appropriate cryo-storage times of SVF-gel as well as formation and absorption rates of transplanted vegetable oil capsules caused by different cryo-storage time. In this study, we analyzed cryopreservation of SVF-gel in a mouse model and compared their tissue activity with different cryopreservation times.

It provides a preliminary basis for discussing the suitable cryopreservation time of SVF-gel.

## Materials and Methods

### Preparation of SVF-Gel

This study was approved by the Ethics Committee of the First Affiliated Hospital of Anhui Medical University (Reference number: Quick -PJ 2020-16-14). Adipocytes were donated from four healthy non-obese female patients, aged 22–40 years, who underwent liposuction surgery in the Plastic Surgery Department of the First Affiliated Hospital of Anhui Medical University. SVF-gel was prepared using a method earlier reported by Professor Lu. After satisfactory swelling and anesthesia, a multi-hole blunt head liposuction needle with a side hole diameter of 1 mm and a needle tube diameter of 3 mm was used to connect a 20 ml syringe to absorb abdominal or femoral fat under negative pressure (kept at about − 0.75 ATM). The fat extract was centrifuged at 1200 g/min for 3 min. The oil and liquid in the upper layer were discarded and the “Coleman fat” in the middle layer retained. A wide-headed straw was used to transfer the upper two-thirds of Coleman fat into a 20 ml tube, which was subsequently defined as low density fat. Another 20 ml syringe was connected by an SVF-gel Luer-lock connector. Next, the low density fat was transferred between two syringes connected to the luer-lock connector at a rate of 20 mL/s(6–8 times) until the low density Coleman fat was transformed into a uniform emulsion. The contents were then centrifuged at 2000 g/min for 3 min. Finally, the middle layer of viscous material, termed the SVF-gel, was collected for further use.

### Cryopreservation and Recovery of the SVF-Gel

The freshly prepared SVF-gel was transferred to a 1 ml syringe, sealed in a sterile bag, and immediately stored at  − 20 °C in a freezer for 5, 15 and 45 days. Frozen SVF-gel was thawed by soaking it in a water bath, at 37 °C for 3 min, with fresh and unfrozen SVF-gel used as controls.

### Specimen Observation and Determination of Volume Retention Rate

SVF-GEL (1 ml) was frozen for 5, 15 and 45 days, respectively, thawed, followed by analysis of color changes and liquefaction by naked eyes. Let sit for 30 min, then use a syringe to suck out the liquid oil droplets. We employed the drainage method to measure the volume of the SVF-gel, after fresh and frozen recovery. These were then used to calculate the rate of retention after freezing.

### TUNEL Assay and Quantification

Fresh SVF-gel and were cryopreserved for 5, 15, and 45 days, preparation of paraffin specimens, SVF-gel with different cryopreservation times from the same donor. 5 µm sections were deparaffinized in xylene and hydrated in graded ethanol. Tissue sections were rinsed with tap water, and then treated with Proteinase K (20 μg/mL in PBS) for 15 min, incubated with Tunel detection solution: TdT enzyme (5 uL) and Fluorescent labeling solution (green, 45 uL) at 37 °C for 60 min. The paraffin-embedded sections were subjected to TUNEL assay using the TUNEL kit, according to the manufacturer’s instructions. Nuclei were stained with DAPI (blue), and the rate of apoptosis is calculated as a percentage of both TUNEL and DAPI-positive nuclei after counting at least 500 cells in five random views (1 × 200 magnification).

### Fat Grafting in an Immunodeficient Mouse Model

Immunodeficient mice were used to establish a human-mouse xenograft model, according to Research Ethics Board-approved protocols. Briefly, 4-week-old male nude mice (BALB/ CAJCL-FOXN1NU/NU) weighing 12–14 g were purchased from Jiangsu Jicui Yaokang Biotechnology Co., LTD. (Nanjing). The mice were administered with general anesthesia, using 1% phenobarbital (50 mg/kg). The mice were then randomized into four groups (6 mice in each group) and injected subcutaneously on both flanks as follows: 0.3 mL of fresh SVF-gel, 5 days cryopreserved SVF-gel, 15 days cryopreserved SVF-gel, and 45 days cryopreserved SVF-gel. Grafts were harvested at 1 month post-operation, weighed, their volumes measured and fixed.

### Histology and Immunofluorescence Staining

The grafts were paraffin-embedded, sectioned into 5 μm slices and stained with hematoxylin eosin (H&E) and Masson trichromatic staining (MT) following standard protocols. The stained sections were then observed and evaluated under an optical microscope (BA410E fluorescence microscope, Motic). For immunofluorescence staining, the sections were dewaxed with dimethylbenzene, hydrated with gradient alcohol, repaired in the citrate repair solution under high pressure and high temperature for 2  min, then cooled at room temperature. The 5-μm-thick sections were blocked for 30 min in goat serum, incubated with primary antibodies at 37 °C for 1 h, namely Guinea pig anti-Perilipin/PLIN1 and rabbit anti-von Willebrand factor. After being washed with PBS, the sections were incubated 30 min at room temperature with secondary antibody (Goat pAb to Rb IgG(CY3), Goat pAb to Ms IgG(FITC), and then washed with PBS. DAPI was used for counterstain. The sections were observed under a fluorescence microscope (BA410E fluorescence microscope, Motic), and images were obtained. Surviving fat cells and vascularization were labeled with Perilipin and vWF, respectively.

### Statistical Analysis

Statistical analysis was performed using SPSS version 25.0 (SPSS Inc.), and data were presented as means ± standard deviations (SD). Data were analyzed with the nonparametric Kruskal–Wallis rank sum test followed by Dunn’s post test. *P *< 0.05 was considered statistically significant.

## Results

### Frozen SVF-Gel has a Lower Volume Retention Rate Compared to Fresh SVF-Gel

**Fig. 1:**
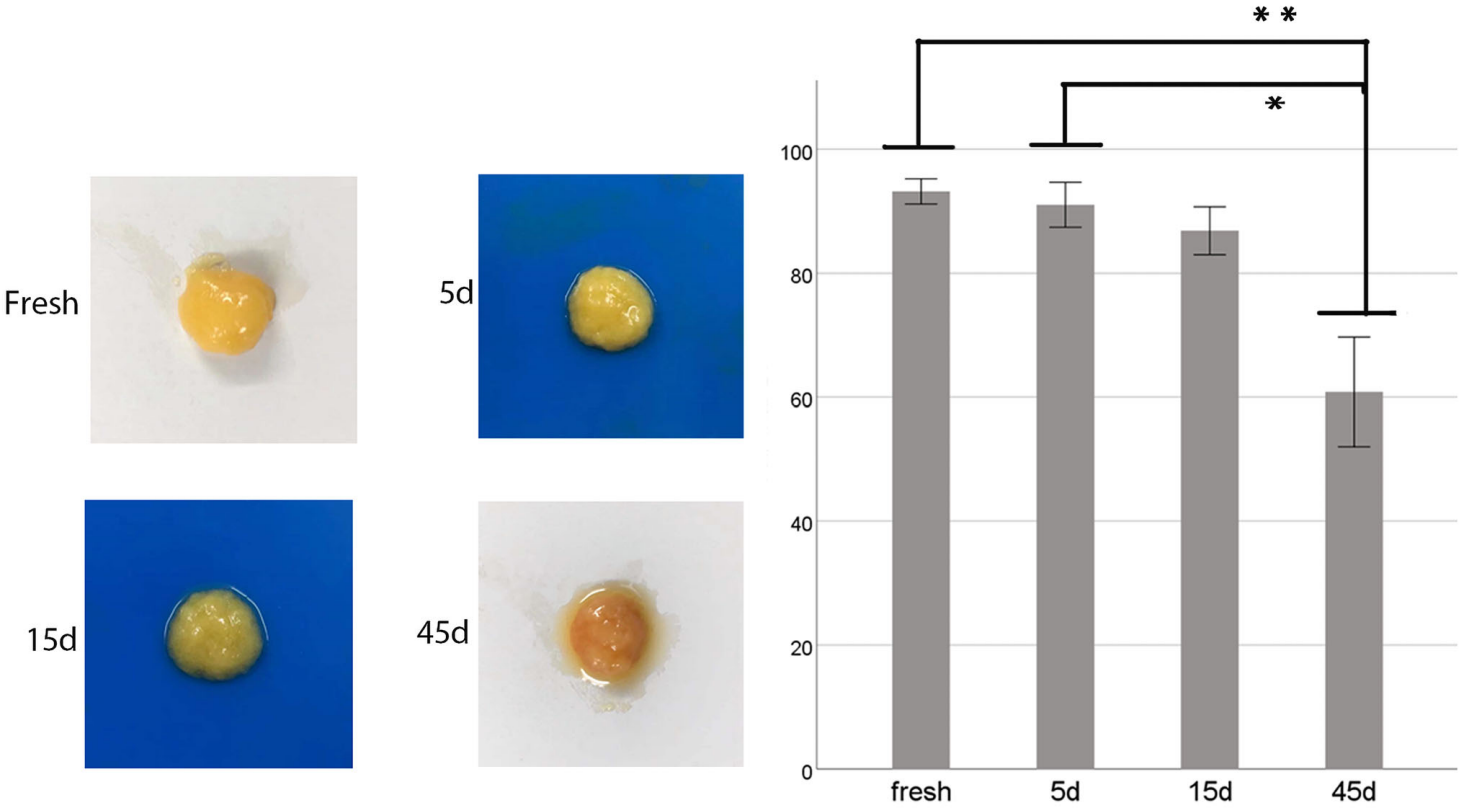
Retention of SVF-gel after thawing. **a**: Fresh SVF-gel appeared gelatinous with a uniform texture. Freeing SVF-gel for 5 days maintained its basic form without oil droplets precipitation. The basic morphology of SVF-gel could still be preserved after cryopreservation for 15 days, and a small amount of oil droplets could be precipitated. SVF-gel cryopreserved for 45 days exhibited a collapsed basic fatty structure with a surface that was covered with released oil. However, some of the original morphology was still retained. **b**: Profiles of volume retention of SVF-gel. After thawing SVF-gel, the oil droplets were removed and the remaining volume was measured. Prolonging freezing time reduced the rate of volume retention of SVF-gel but increased the rate of oil droplet precipitation. After 45 days of cryopreservation, the SVF-gel volume retention rate was 60.8 ± 8.4%. The volume retention rate at 5 days of cryopreservation and 15 days of cryopreservation group was not significantly decreased compared with that of fresh SVF-gel. *p = 0.014, **p = 0.012. Scale bar = 50 μm. *n *= 6

The SVF-gel samples frozen for 5 days, 15 days, and 45 days were thawed for 3 min, and then, the frozen SVF-gel and fresh SVF-gel 0.3 ml were injected into the background plate. Analysis of SVF-gel, 1 min after injection into a background plate, revealed that those frozen for 5 days still maintained their basic morphology, with no evidence of oil droplet precipitation, compared to fresh SVF-gel. Similarly, those frozen for 15 days also retained their basic morphology, although they exhibited a small amount of oil droplet precipitation. Conversely, SVF-gel frozen for 45 days exhibited a collapsed basic fat structure, although part of its original form was retained, and also showed precipitation of a large number of oil droplets (Fig.1a). Next, we measured 1 ml fresh and cryopreserved SVF-gel thawed to remove the precipitated oil droplets. Results revealed that the three groups of freeze-thawed SVF-gel had lower survival rates than fresh ones. The volume retention rate of oil droplets removed by fresh SVF-gel was 93.2 ± 1.9%, while those frozen for 5 and 15 days were 91 ± 3.5% and 86.8 ± 3.7%, respectively, after thawing. We found no statistically significant differences in volume retention rates between fresh and frozen SVF-gel at 5 and 15 days. SVF-gel frozen for 45 days had a volume retention rate of 60.8 ± 8.4%, which was significantly lower than that in the control group (fresh SVF-gel), (*P *= 0.012). Compared with the SVF-gel group frozen for 5 days, it also decreased significantly (*P *= 0.014) (Fig. 1b).

### Freezing time Affected Cell Death Associated with Frozen SVF-Gel

**Fig. 2:**
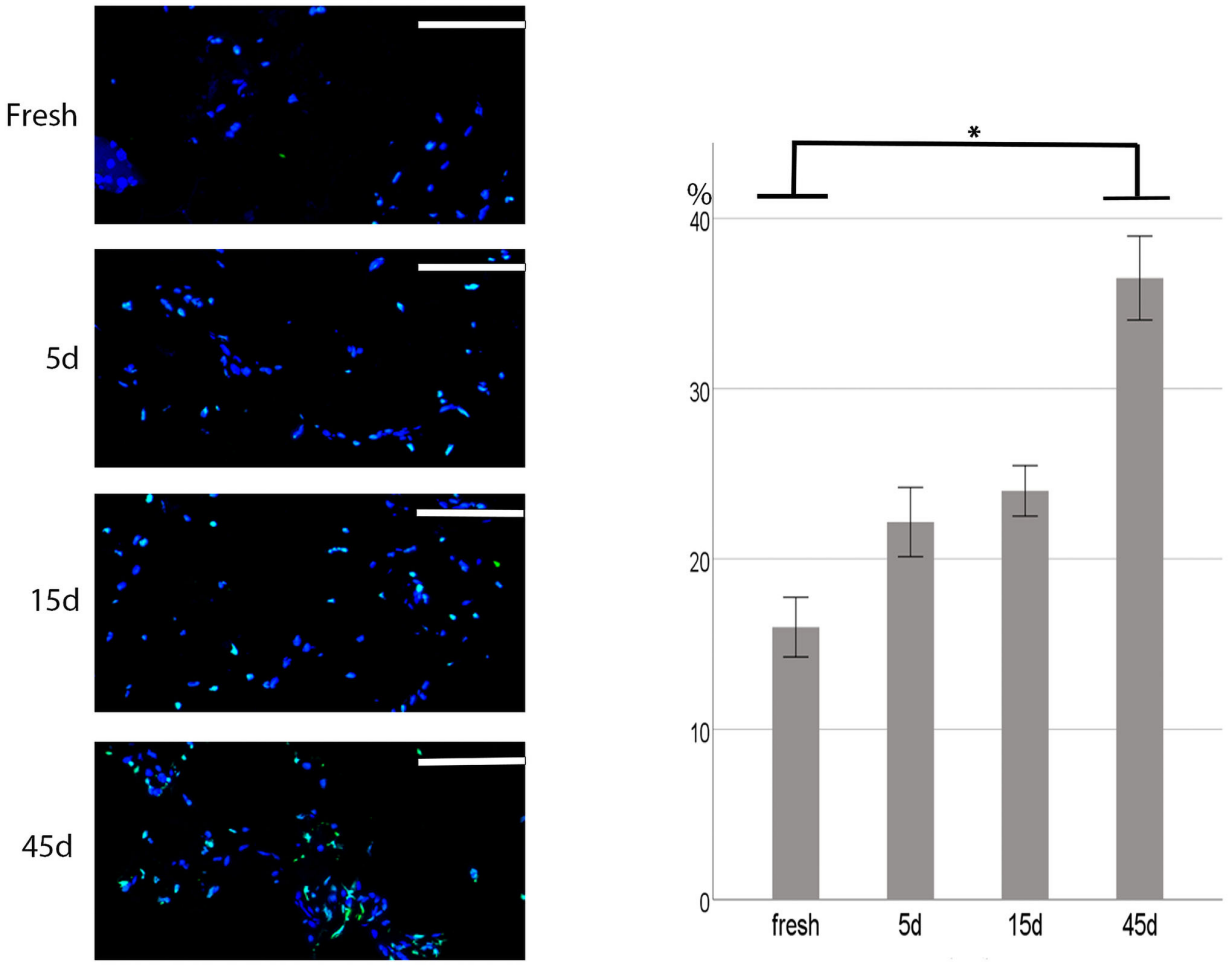
TUNEL analysis of frozen tissue sections. Frozen sections of SVF-gel in control group (fresh SVF-gel), 5-days cryopreservation group, 15-days cryopreservation group, and 45-days cryopreservation group. The TUNEL assay was performed to determine the cell apoptosis rate. Prolonged cryopreservation time increased apoptosis of SVF cells. The apoptosis rate was 36.5 ± 2.3% after 45 days of cryopreservation. *p < 0.01. Scale bar = 50 μm. *n *= 6

Next, we performed a TUNEL assay to analyze freeze-induced cell death between frozen and control SVF-gel (Fig. 2). Results showed that the apoptosis rate of fresh SVF-GEL cells was 16 ± 1.7%. We found no statistically significant differences in cell viability between SVF-gels cryopreserved for 5 days at − 20 °C with fresh ones. The apoptosis rate for the 5 days group was 22.2 ± 1.9%. SVF-gel cryopreserved for 15 days had an apoptosis rate of 24 ± 1.4%, which was higher but not significantly different from than that of fresh SVF-GEL. SVF-GEL cryopreserved for 45 days had an apoptosis rate of 36.5 ± 2.3%, which was significantly higher than that of fresh SVF-GEL (*P *= 0.005) (Fig. 2).

### Prolonged Freezing Time Reduced the Graft Retention Rate

**Fig. 3:**
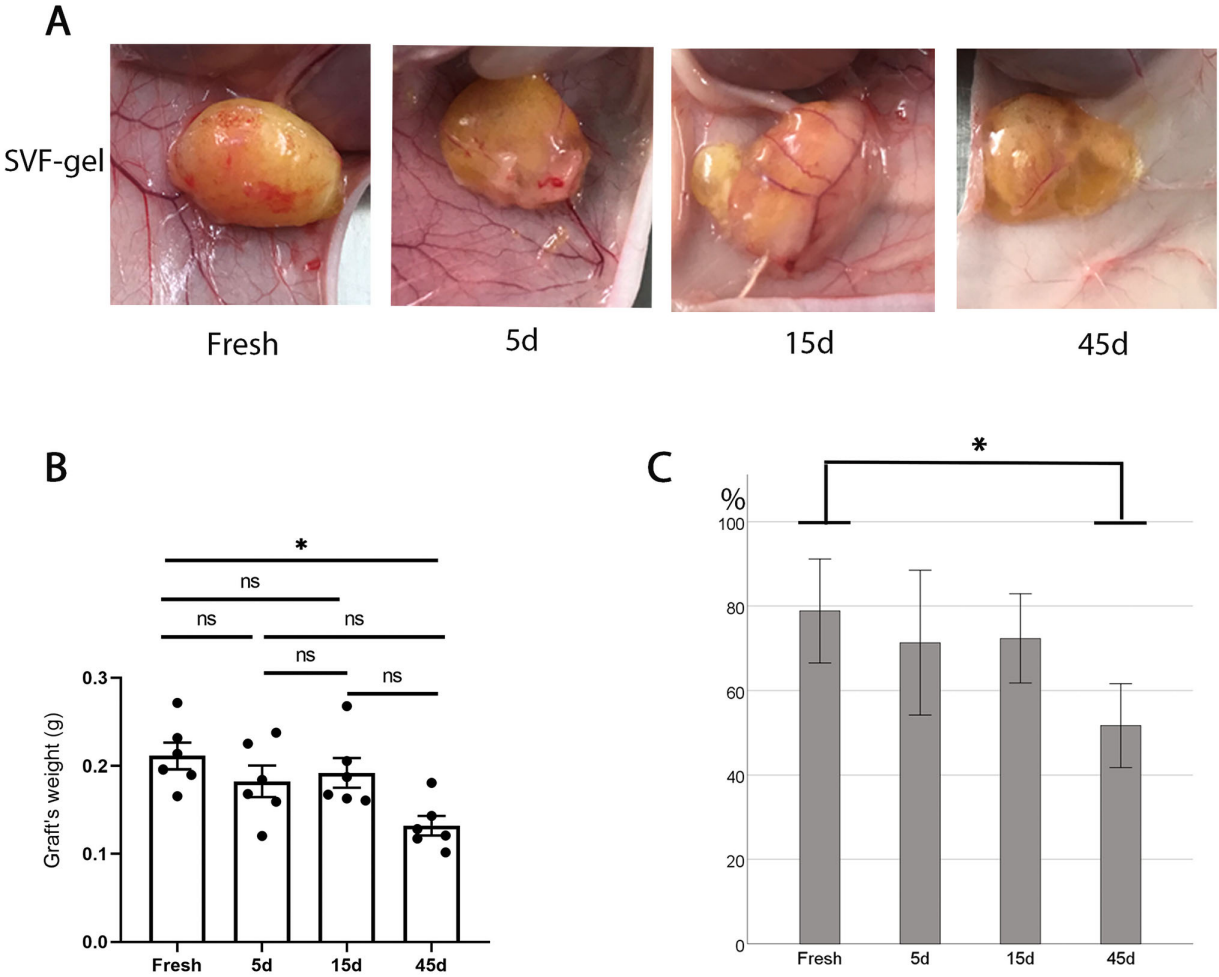
Retention after SVF-gel transplantation. **a** Gross appearance of a representative SVF-gel graft. The SVF-gel and fresh SVF-GEL with different cryopreservation durations were transplanted subcutaneously into mice, and samples were taken one month later. **b** Quality retention of the graft in the fresh SVF-gel group showed better weight maintenance than the 45-days cryopreservation group. The quality of the early grafts decreased only slightly with the extension of cryopreservation time, while the quality of the grafts decreased significantly with the extension of cryopreservation time to 45 days (*P *= 0.012). *n*=6. **c** The fresh SVF-gel group showed better volume retention compared to the 45-days frozen storage group. The volume of the grafts decreased slightly with the extension of cryopreservation time, but decreased significantly with the extension of cryopreservation time to 45 days (*P *= 0.014). *n*=6

We subcutaneously transplanted fresh and cryopreserved SVF-gel (frozen for 5, 15 and 45 days) into nude mice, then collected and analyzed grafts 1 month after surgery. A summary of the graft is illustrated in Fig. 3a. Summarily, fresh SVF-gel exhibited a soft and elastic graft surface, with good blood vessel formation around the graft and no obvious oil sac formation. After 5 days of cryopreservation, SVF-gel grafts still maintained good tissue integrity, and neovascularization could be observed on and around the grafts. SVF-gel grafts cryopreserved for 15 days showed oil sacs, with minimal surface angiogenesis relative to those in the control group. However, SVF-gel frozen for 45 days exhibited markedly large oil sacs on the grafts, with only a small number of new vessels evident. Fresh SVF-gel had better quality and volume retention rates than those across the three cryopreservation groups. Notably, fresh SVF-gel had significantly higher graft quality than those frozen for 45 days (*P *= 0.012). However, SVF-gel frozen for 5 and 15 days had a slightly lower graft quality than fresh ones (Fig. 3b). Moreover, fresh SVF-gel had a volume retention rate of 79 ± 12%, which decreased to 71 ± 16% after 5 days of cryopreservation. Cryopreservation for 15 days reduced the volume retention rate of the SVF-gel to 72 ± 10%. However, freezing the SVF-gel for 45 days resulted in a significantly lower rate of volume retention (52 ± 9%) relative to the control group (*P *= 0.014) (Fig.3c).

### Longer Freezing Time Resulted in Impaired Histological Structure in SVF-Gel Grafts

**Fig. 4:**
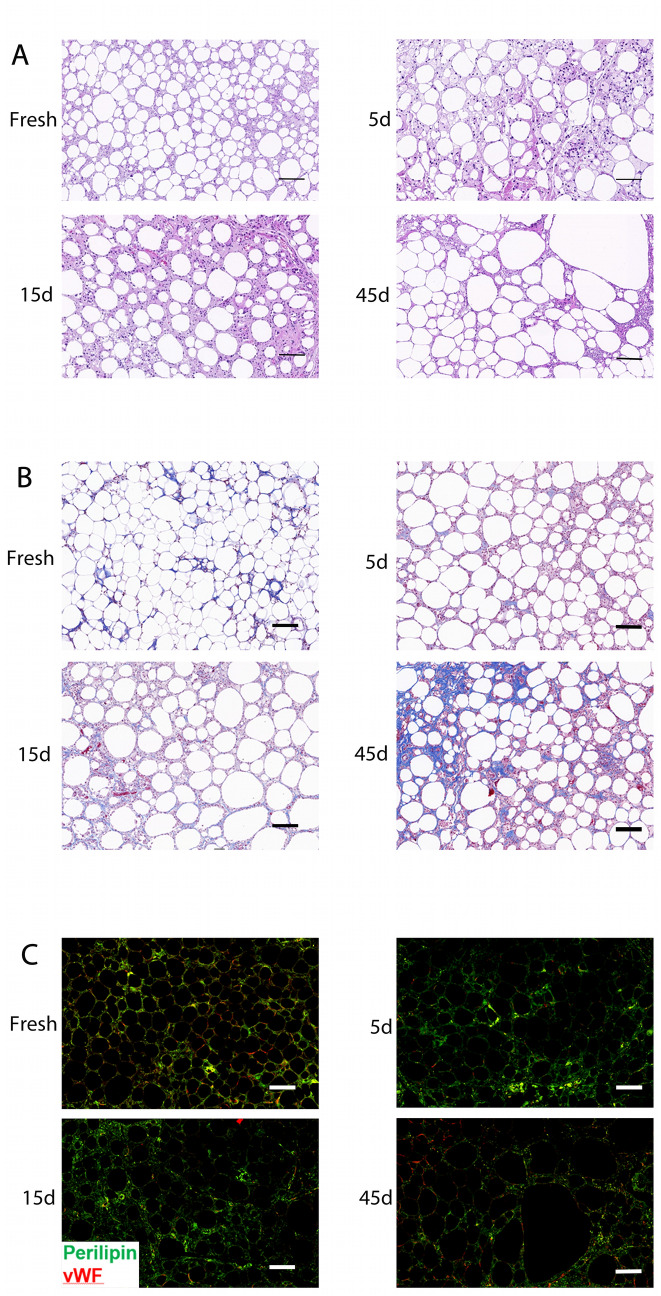
Histological evaluation of the graft. **a** H&E staining. There were fewer oil droplets in the fresh SVF-gel transplantation group than in the frozen gel transplantation group. Increasing the cryopreservation time resulted in the formation of more fat necrosis and oil sacs, but this was accompanied by a reduction in the number of mature fat cells. **b** Masson trichromatic staining results. Grafts in the fresh SVF-gel group showed reduced proliferation of fibrous connective tissue compared with those in the cryopreservation group. Prolongation of the freezing time increased the number of blue-stained collagen fibers. Grafts in the 45-days cryopreservation group had a higher number of proliferative fibrous connective tissue. **c** Immunofluorescence staining results showing the number of surviving fat cells which were labeled with Perilipin and vascularization was labeled with vWF. Fresh SVF-gel grafts showed Perilipin + mature adipose tissue. The number of healthy adipose tissues and angiogenesis were gradually decreased with the increase in freezing time. Scale bar = 50 μm. *n*=6

HE staining of the grafts showed that the adipose tissue after fresh SVF-gel transplantation was orderly and had a complete shape, with no evidence of obvious oil sac formation and minimal proliferation of the fibrous connective tissue. Cryopreserving the specimens for 5 days showed that the SVF-gel grafts still maintained a good arrangement structure of adipose tissue. However, the adipose cells had a relatively loose arrangement. In addition, there were still some undifferentiated adipose precursor cells between the adipose cells, and a small amount of fibrous connective tissue could be seen at the edge of the graft specimen. SVF-gel grafts cryopreserved for 15 days still had a certain adipose arrangement structure. Notably, grafts in this group were loosely arranged, some undifferentiated adipose precursor cells and small oil sacs could still be seen between the adipose cells, with fibrous connective tissue interspersed between the tissues, relative to fresh ones. However, SVF-gel grafts cryopreserved for 45 days exhibited a disorganized adipose tissue structure, with small oil sacs fusing into large ones (Fig. 4a). Marson’s trichromatic staining also revealed proliferation of a large amount of fibrous connective tissue after 45 days of cryopreservation. However, grafts of fresh SVF-gel, as well as those cryopreserved for 5 and 15 days, exhibited littles blue-stained collagen fibers (Fig. 4b). Immunofluorescence results showed that the grafts in the fresh SVF-gel group had uniform Perilipin + living fat cells, while those cryopreserved for 45 days had significantly fewer Perilipin + living fat cells. Perilipin + living fat cells were still evident after transplantation of SVF-gel frozen for 5 and 15 days. Moreover, their Perilipin + living fat cells were loosely arranged compared to those of fresh SVF-gel (Fig. 4c).

## Discussion

Cryopreservation of the adipose tissue, as an ideal method of adipose tissue preservation, has generated numerous research attention. Lu [5] demonstrated that SVF-gel made by mechanical methods selectively destroyed mature fat cells, thereby releasing about 80% of fat oil. Low oil and deep cryopreservation of SVF-gel can reduce graft complications associated with adipocyte necrosis. In the SVF-gel cryopreservation model established by his team, SVF-gel showed better cryopreservation potential than liposuction solution, as well as short-term preservation potential after transplantation [20]. Previous studies have shown that cryopreserved SVF-gel has advantages over traditional adipose tissue under similar conditions. However, the cryopreservation capacity of SVF-gel at different cryopreservation duration has not been clarified. In the present study, we explored whether freezing time could affect cryopreservation of SVF-gel. Macrae noted that there was only a little cryoinjury to adipose tissue cryopreserved at − 20 °C for a certain amount of time, without controlling the rate of freezing and without the use of cryoprotectants [14]. Considering that in clinical use, difficult to obtain laboratory cryopreservation conditions. We need economical and convenient cryopreservation methods to meet the needs of our patients, so simple freezing at  − 20 °C is our first choice. And the thermal stress induces post-thawing cell death [21], to reduce the time of thermal effect during thawing, we choose  − 20 °C Our results demonstrate gross and histological differences among SVF-gel under different cryopreservation times. In so-doing, they provide a basis for determining the maximum cryopreservation time to reduce the potential risk of surgery, ensure the postoperative effect and reduce postoperative complications.

In this study, we froze SVF-gel for 5, 15 and 45 days and found that short-term cryopreservation (5 and 15 days) resulted in no statistically significant differences in volume survival rate and apoptosis rate relative to fresh SVF-gel. However, cryopreservation for 45 days resulted in markedly higher apoptosis rate and lower rate of volume retention in SVF-gel relative to the control. Results from animal models transplanted with SVF-gel showed that those preserved for 45 days also had significantly lower rates of graft volume retention relative to controls. However, no significant differences were recorded between SVF-gel cryopreserved for 5 and 15 days relative to the control group. Immunohistochemical results also showed that SVF-gel frozen for 45 days had worse biological effects on oil sac complications, fat cell regeneration, and vascularization. Collectively, these results demonstrated that short-term cryopreservation of SVF-gel can still guarantee its good transplantation effect, and the increase in short-term cryopreservation time does not bring about great difference in transplantation effect and increase in complications. However, extending the cryopreservation time to 45 days resulted in a relatively poor transplant effect.

Previous studies have shown that most cells from fat aspirates, even those with minor damage, die within the first 1–2 days of cryopreservation [22]. Experimental results from this study showed that short-term cryopreservation (5 and 15 days) has little effect on the rate of cell apoptosis, which further confirmed that SVF-gel had better cryopreservation effect compared with adipose tissue. The difference in transplantation effect between SVF-gel cryopreserved for 45 days and those at 5 and 15 days may be attributed to the cell preservation ability of the cryopreservation gel. The cryopreservation group with higher apoptosis rate for 45 days showed poor graft effect.

A previous study demonstrated that cryopreservation of adipose-derived stem cells for 1 month had little effect on their ability to differentiate into mature adipocytes, while ASCs cells still retained a considerable degree of differentiation potential [17]. Fibronectin is an important periadipocyte ECM protein that regulates cell shape and adipogenic gene expression in adipocytes [23, 24]. Type IV collagen participates in the formation of periadipocyte basement membrane [25]. Previous studies have shown that cryopreservation of adipose tissue at − 20 °C mediates a gradual loss of type IV collagen and fibronectin, SVF-gel contains more ECM protein than fat aspirate, and the contents of type IV collagen and fibronectin in SVF-gel remain high after one month of freezing [20]. This may explain the lack of significant differences in experimental results between SVF-gel frozen for 5 and 15 days relative to fresh ones. Moreover, we observed little fluctuation in the quality and volume of SVF-gel frozen for 5 and 15 days relative to controls, albeit at no statistical significance. These results suggested that SVF-gel had similar cell activity after cryopreservation for 5 days and cryopreservation for 15 days, consistent with results from previous in vitro experiments [20].

Niche (stem cell niche), which refers to the microenvironment surrounding stem cells (including adjacent ECM, cells and adhesion molecules of stem cells), can regulate the behavior of stem cells through different signals. Previous studies have demonstrated the importance of the interaction between stem cells and Niche in proliferation, self-renewal and differentiation of stem cells. Moreover, stem cells lacking Niche protection will be rapidly depleted [26, 27]. Therefore, SVF-gel, which is composed of a large number of ECM/SVF particles, has been implicated in retention of a good Niche structure. We speculated that type IV collagen and fibronectin may have been gradually lost in SVF-gel after 45 days of cryopreservation. The decrease in the protective capacity of Niche on stem cells suppresses the proliferation and differentiation ability of stem cells leading to a decline in the amount of secreted VEGF, HGF, and other angiogenic factors, as well as the reduced angiogenesis in the graft. This may explain the cell apoptosis rate and graft results of SVF-gel after cryopreservation for 45 days. We found that after 45 days of SVF-gel cryopreservation, graft vascularization was reduced. Vascular smooth muscle cells (VSMCs) play essential roles in regulating blood vessel form and function and they are required for vascular tissue regeneration [28]. Xiaoqing Zhang found that ASCs have reduced potential for differentiation to VSMC-like cells after one month of cryopreservation, the VSMC-like cells derived from cryopreserved ASCs expressed smoothelin gene and protein at lower levels. The results of this in vitro experiment may explain the phenomenon of vascularization of grafts.

The freezing time of SVF-gel in this experiment, as well as the increase in the rate of apoptosis, and deteriorated graft effect of SVF-gel following extension of freezing time require further research exploration. Follow-up studies are expected to identify indicators of the two groups before and after the experimental critical point. Detection of cell metabolites and harmful indicators is expected to affirm the significance of freezing time, in order to make a convenient and effective prediction of frozen graft activity in clinic.

## Conclusion

In summary, these results indicate that freezing time can affect the transplantation effect of SVF-gel to a certain extent. Short-term cryopreservation of SVF-gel at − 20 °C (for 5 and 15 days) has no marked effect on both cell viability and the rate of apoptosis. However, extending cryopreservation time (to 45 days) resulted in increased apoptosis rate and more negative transplantation effect. Collectively, these results not only provide a preliminary basis for future optimization of cryopreservation time of SVF-gel, but also set up a platform for further animal experiments on clinical cryopreservation use of SVF-gel.

## References

[1] Coleman SR (2001). Structural fat grafts: the ideal filler?. Clin Plast Surg.

[2] Khouri RK, Khouri RK (2017). Current clinical applications of fat grafting. Plast Reconstr Surg.

[3] Suga H, Eto H, Aoi N (2010). Adipose tissue remodeling under ischemia: death of adipocytes and activation of stem/progenitor cells. Plast Reconstr Surg.

[4] Doi K, Ogata F, Eto H (2015). Differential contributions of graft-derived and host-derived cells in tissue regeneration/remodeling after fat grafting. Plast Reconstr Surg.

[5] Yao Y, Dong Z, Liao Y (2017). Adipose extracellular matrix/stromal vascular fraction gel: a novel adipose tissue-derived injectable for stem cell therapy. Plast Reconstr Surg.

[6] Zhang Y, Cai J, Zhou T, Yao Y, Dong Z, Lu F (2018). Improved long-term volume retention of stromal vascular fraction gel grafting with enhanced angiogenesis and adipogenesis. Plast Reconstr Surg.

[7] Yao Y, Cai J, Zhang P (2018). Adipose stromal vascular fraction gel grafting: a new method for tissue volumization and rejuvenation. Dermatol Surg.

[8] Ohashi M (2020). Fat grafting for facial rejuvenation with cryopreserved fat grafts. Clin Plast Surg.

[9] Ohashi M, Chiba A, Nakai H, Fukuda E, Higuchi T (2018). Serial injections of cryopreserved fat at − 196 °C for tissue rejuvenation, scar treatment, and volume augmentation. Plast Reconstr Surg Glob Open.

[10] Yang HJ, Kang SY (2020). Comparisons between fresh and cryopreserved fat injections in facial lipofilling. Arch Craniofac Surg.

[11] Shu Z, Gao D, Pu LL (2015). Update on cryopreservation of adipose tissue and adipose-derived stem cells. Clin Plast Surg.

[12] Varghese J, Mosahebi A (2017). Historical overview of stem cell biology and fat grafting. Aesthet Surg J.

[13] Pu LL (2009). Cryopreservation of adipose tissue. Organogenesis.

[14] MacRae JW, Tholpady SS, Ogle RC, Morgan RF (2004). Ex vivo fat graft preservation: effects and implications of cryopreservation. Ann Plast Surg.

[15] Mashiko T, Wu SH, Kanayama K (2018). Biological properties and therapeutic value of cryopreserved fat tissue. Plast Reconstr Surg.

[16] Miyagi-Shiohira C, Kurima K, Kobayashi N (2015). Cryopreservation of adipose-derived mesenchymal stem cells. Cell Med.

[17] Goh BC, Thirumala S, Kilroy G, Devireddy RV, Gimble JM (2007). Cryopreservation characteristics of adipose-derived stem cells: maintenance of differentiation potential and viability. J Tissue Eng Regen Med.

[18] Gonda K, Shigeura T, Sato T (2008). Preserved proliferative capacity and multipotency of human adipose-derived stem cells after long-term cryopreservation. Plast Reconstr Surg.

[19] Robinson PG, Murray IR, West CC (2019). Reporting of mesenchymal stem cell preparation protocols and composition: a systematic review of the clinical orthopaedic literature. Am J Sports Med.

[20] Feng J, Hu W, Fanai ML (2019). Mechanical process prior to cryopreservation of lipoaspirates maintains extracellular matrix integrity and cell viability: evaluation of the retention and regenerative potential of cryopreserved fat-derived product after fat grafting. Stem Cell Res Ther.

[21] Ma XH, Shi Y, Hou Y (2010). Slow-freezing cryopreservation of neural stem cell spheres with different diameters. Cryobiology.

[22] Son D, Oh J, Choi T (2010). Viability of fat cells over time after syringe suction lipectomy: the effects of cryopreservation. Ann Plast Surg.

[23] Spiegelman BM, Ginty CA (1983). Fibronectin modulation of cell shape and lipogenic gene expression in 3T3-adipocytes. Cell.

[24] Singh P, Carraher C, Schwarzbauer JE (2010). Assembly of fibronectin extracellular matrix. Annu Rev Cell Dev Biol.

[25] Lin D, Chun TH, Kang L (2016). Adipose extracellular matrix remodelling in obesity and insulin resistance. Biochem Pharmacol.

[26] Moore KA, Lemischka IR (2006). Stem cells and their niches. Science.

[27] Scadden DT (2006). The stem-cell niche as an entity of action. Nature.

[28] Zhang X, Simmons CA, Santerre JP (2018). Alterations of MEK1/2-ERK1/2, IFN*γ* and Smad2/3 associated signalling pathways during cryopreservation of ASCs affect their differentiation towards VSMC-like cells. Stem Cell Res.

